# A Brief History of the Use of Insecticides in Brazil to Control Vector-Borne Diseases, and Implications for Insecticide Resistance

**DOI:** 10.3390/tropicalmed10120336

**Published:** 2025-11-27

**Authors:** Bashir Alsharif, Maria Alice Varjal Melo-Santos, Rosângela Maria Rodrigues Barbosa, Constância Flávia Junqueira Ayres

**Affiliations:** 1 Department of Entomology, Aggeu Magalhães Institute-Fiocruz, Recife 50740-465, Brazil; bashiralsharif@gmail.com (B.A.); maria.varjal@fiocruz.br (M.A.V.M.-S.); rosangela.barbosa@fiocruz.br (R.M.R.B.); 2 Department of Medical Entomology, Ministry of Health, Khartoum 11111, Sudan

**Keywords:** vector control, insecticide resistance, *Aedes aegypti*, *Anopheles* spp., *Culex quinquefasciatus*, triatomine, phlebotomine

## Abstract

In Brazil, public health programs have relied predominantly on chemical insecticides to control *Aedes aegypti*, *Anopheles* spp., *Culex quinquefasciatus*, triatomines, and phlebotomines. Rising vector-borne disease incidence and insecticide resistance (IR) call for a critical appraisal of historical and current control practices. This literature review compiles secondary data produced from 1901 to 2024 obtained from Medline/PubMed, Google Scholar, and governmental notes and reports. Brazil’s vector control progressed from organochlorines (e.g., DDT) to organophosphates, carbamates, pyrethroids, insect growth regulators, microbial larvicides (Bti and Lsp), spinosad, and recently formulations with dual active-ingredient. *Ae. aegypti* showed widespread resistance to temephos and pyrethroids, decreased susceptibility to pyriproxyfen, and no documented Bti resistance. *Anopheles* spp. exhibited low to moderate resistance to pyrethroids. *Cx. quinquefasciatus* resistance is likely influenced by collateral exposure from *Aedes* control and domestic use. Regarding triatomines and phlebotomines, there was a predominant reliance on pyrethroids; most studies indicate their susceptibility to these compounds. In short, Brazil’s century-long, insecticide-centric strategy has delivered episodic gains but fostered *Aedes aegypti* resistance. For other species, for which there is no dedicated program for a long period, data on resistance are scarce or nonexistent. Sustainable progress requires strengthened, nationwide IR surveillance and entomological mapping to coordinate cross-program actions.

## 1. Introduction

Vector-borne diseases (VBDs) are infections caused by pathogens transmitted by arthropods, such as mosquitoes (dengue, yellow fever, chikungunya, Zika, Mayaro fever, Rift Valley fever, malaria, West Nile fever, Japanese encephalitis, and lymphatic filariasis); sand flies (leishmaniasis); triatomine bugs (Chagas disease); blackflies (onchocerciasis); fleas (plague and tungiasis); lice (typhus, louse-borne relapsing fever); tsetse flies (human African trypanosomiasis); ticks (Lyme disease, relapsing fever and Crimean-Congo hemorrhagic fever); and biting midges (culicoides-borne viral diseases and Oropouche fever). Each year, over 700,000 deaths are caused by vector-borne diseases, with mosquitoes having the most significant epidemiological impact. More than 80% of the world’s population lives in areas at risk of transmission of these diseases [1,2,3].

**Figure 1:**
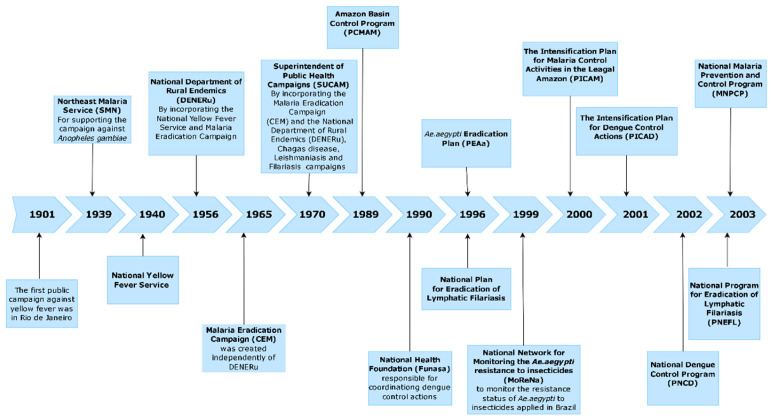
Timeline of the most important Brazilian national public health programs.

Vaccines are unavailable for the majority of these VBDs, except for yellow fever [4] and Japanese encephalitis [5]. Recently, vaccines have become available for dengue, such as Dengvaxia^®^, TV003/TV005, TAK-003 (QDENGA), and Butantan-DV, and for Chikungunya [6,7]. Vaccines for Zika are still under clinical trials [8,9,10], but face numerous challenges, such as the lack of well-characterized pregnancy models of ZIKV infection, the possibility of cross-reactive antibodies to worsen symptoms of DENV infection, and the difficulty of estimating their efficacy without ongoing transmission in endemic areas [11]. Unfortunately, we have witnessed a growing spread of these diseases to new areas and, at the same time, a significant increase in the number of cases in endemic areas for some of these diseases. This has jeopardized the goals of VBD elimination programs and left authorities responsible for controlling these diseases without good prospects. Additionally, predictive models regarding the impact of global warming on the increase in the incidence of these diseases in future scenarios underscore the seriousness of the situation [12]. Figure 1 shows the timeline of the most important Brazilian national public health programs, targeting VBD.

Vector control remains the primary method for preventing VBDs, aiming to reduce the population density of target species involved in pathogen transmission and limit human exposure. Vector control approaches can be grouped into chemical and non-chemical methods (physical-mechanical, genetic, biological, and behavioral) [3]. Despite the numerous technologies that have emerged for mosquito control, such as the use of *Wolbachia*, sterile and transgenic mosquitoes, larvicide dissemination stations, and toxic sugar baits, among others, the advances in these methodologies are still under evaluation for efficacy and sustainability in some countries, including Brazil [3,13]. In addition, cost is another major reason why chemical compounds remain popular compared to other methods. Therefore, the use of chemical insecticides, in practice, remains the major pillar of vector control.

Insecticides are classified according to their chemical composition into inorganic, organic, and synthetic organic [14]. They can also be categorized into larvicides and adulticides based on the targeted stage of the vector’s life cycle. Historically, numerous important national public health programs and campaigns have been launched to address public health emergencies in Brazil (Figure 1). Most of these campaigns adopted insecticides as a method of vector control. This study aims to summarize the historical and current situation of the application of insecticides by the Ministry of Health in Brazil. This work is an update that tracks the use of insecticides adopted for the control of *Ae. aegypti*, *Anopheles*, *Culex quinquefasciatus*, Phlebotomine, and triatomine vectors, and their implications on the development of insecticide resistance in natural populations of these species in Brazil.

## 2. Materials and Methods

Secondary data regarding the use of insecticides for vector control from 1901 to 2024 was obtained from scientific publications in Medline/PubMed, Google Scholar database, technical notes, and national reports, with the following Medical Subject Headings (MeSH) terms: (1) Vector control AND Insecticide AND Aedes AND Brazil; (2) Vector control AND Insecticide AND Anopheles AND Brazil; (3) Vector control AND Insecticide AND Culex AND Brazil; (4) Vector control AND Insecticide AND Triatomine AND Brazil; (5) Vector control AND Insecticide AND Phlebotomines OR sandfly AND Brazil. In addition, the terms insecticide resistance AND each vector name (*Aedes aegypti*, *Culex quinquefasciatus*, *Anopheles* spp., triatomine, and phlebotomine) AND Brazil were used to select relevant papers about reports of insecticide resistance in these species. Equivalent search terms were also used in Portuguese.

## 3. Results

### 3.1. Aedes aegypti Linnaeus, 1762

*Aedes aegypti* is widely distributed across Brazil and is the primary vector for several arboviral diseases. This species exhibits both anthropophilic and endophilic behaviors, reflecting its high contact with human hosts [15]. This mosquito species is the only one for which the Brazilian government has implemented nationwide control programs, which have been adapted over the years and remain in place to the present. This stems from its historical role as a vector of yellow fever and its main role in the triple epidemic of DENV, ZIKV, and CHIKV.

Interestingly, historical vector control efforts against *Ae. aegypti* dates back centuries. Even before the introduction of DENV in Brazil, *Ae. aegypti* was already considered a problem due to its involvement in the transmission of urban yellow fever (YF). The first epidemic of YF in Brazil occurred in Recife, the capital of the state of Pernambuco, in 1685. The first prophylactic campaign took place in 1691, led by João Ferreira da Rosa, a clinician from Portugal. Notably, these measures were not intended specifically for the vector, as the role of *Ae. aegypti* as the vector for urban yellow fever had not yet been identified. Prophylactic measures at the time included lighting bonfires, fumigating dwellings, cleaning streets, and environmental management [16].

In 1881, Cuban clinician Carlos J. Finlay proposed a theory regarding the role of mosquitoes in the transmission cycle of yellow fever. This theory was later confirmed in 1901 by Walter Reed. During the yellow fever epidemic in São Paulo from 1901 to 1903, Emilio Ribas used kerosene and petroleum oil derivatives as larvicides, along with sulfur and pyrethrum for indoor fumigation against adult *Ae. aegypti*. This method was later adopted by Oswaldo Cruz during the 1903–1909 yellow fever outbreak in Rio de Janeiro, where he used a mixture of kerosene and creolin with eucalyptus oil as a larvicide [16]. From 1928 to 1929, during the second outbreak of yellow fever in Rio de Janeiro, various insecticides were used, including pyrethrum, xylene, cresol, methyl salicylate in kerosene, and carbon tetrachloride (CCl_4_) for controlling adult mosquitoes; petroleum derivatives were used as larvicides. Generally, between 1901 and 1946, petroleum derivatives, pyrethrum, and inorganic substances were primarily used to control *Ae. aegypti* [16,17].

The global introduction of the organochlorine insecticide dichlorodiphenyltrichloroethane (DDT) in the 1940s revolutionized vector control efficiency by drastically reducing the transmission of vector-borne diseases such as malaria and typhus [18]. In 1947, the National Service of Yellow Fever in Brazil adopted DDT as the insecticide of choice [16]. In 1955, the Ministry of Health declared Brazil free of *Ae. aegypti* when DDT was considered the “silver bullet” for vector control. In 1958, Brazil, along with countries such as Bolivia, British Honduras, and others, was officially declared free of *Ae. aegypti* at the Pan American Sanitary Conference in Puerto Rico [19].

However, *Ae. aegypti* reinfested Brazil in 1967, with the first reports coming from Belém and later from other Brazilian states. Due to resistance to DDT, the National Department of Rural Endemics (DENERu) began using the organophosphates temephos and fenthion for focal and perifocal control, respectively. Following the depletion of Fenthion stock, it was subsequently substituted by fenitrothion (an organophosphate) as adulticide in 1970 [16,20,21]. In 1973, Brazil declared *Ae. aegypti* eradicated for the second time; however, reinfestation occurred just three years later, spreading throughout the country [22].

Malathion, an organophosphate (OP), was used from 1985 to 1989 as both a larvicide and an adulticide in certain areas of Brazil [23]. It is noteworthy that the first dengue epidemic in Brazil occurred in Rio de Janeiro in 1986, after which it rapidly spread to other regions of the country. A total of 732 confirmed cases were reported following the isolation of Dengue virus type 1 strains from both patients and adult *Ae. aegypti* mosquitoes [24]. The carbamate propoxur was also used in São Paulo State from 1986 to 1989 [25]. From 1989 to 2000, the OPs fenitrothion and malathion were reintroduced [23]. In 2003, deltamethrin (PY) replaced cypermethrin as the adulticide for residual spraying [26].

In 1996, the *Aedes aegypti* Eradication Plan (PEAa) was established. However, the extensive use of temephos and malathion as larvicide and adulticide, respectively, led to a significant reduction in their effectiveness. Consequently, several studies were conducted to evaluate the susceptibility of *Ae. aegypti* populations across different regions of Brazil [25,26,27]. Further studies revealed resistance to both organophosphates and cypermethrin [28], leading to modifications of the PEAa and its version, the PIACD, in 2001. The failure of both programs led to the creation of the National Dengue Control Program (PNCD) in 2002, with the same strategies for mosquito control, but with the aim of controlling the density of the *Ae. aegypti* populations throughout the national territory to prevent dengue outbreaks. In 1999, the National Network for Monitoring *Ae. aegypti* Resistance to Insecticides (MoReNAa) was established by the Brazilian Ministry of Health (MoH) in coordination with the PNCD to monitor the susceptibility of wild *Ae. aegypti* populations in Brazil [29]. The main objective of this network was to characterize the resistance status and underlying mechanisms in Brazilian *Ae. aegypti* populations, while supporting evidence-based decision-making in vector control.

In response to the detection of resistance to temephos, the biolarvicide *Bacillus thuringiensis* var. *israelensis* (Bti) was introduced in 2001 in selected cities, where the resistance ratio (RR_95_) was >10-fold according to the criteria of Mazzari and Georgiou [30]. The use of Bti remained sporadic until 2023, when it was recommended for national use. The insecticidal effect of Bti against mosquito larvae is based on the activity of crystals, which are constituted by multiple protoxins that bind to different and specific receptors in the mosquito midgut [31]. The presence of multiple toxins hinders the emergence of resistance to this entomopathogenic bacterium [32,33].

In the perspective of management of insecticide resistance, the experiments by Melo-Santos et al. [31] showed that temephos susceptibility could be restored in a resistant strain of *Ae. aegypti*. However, the process of reversing resistance could be very slow and lengthy, due to its multifactorial inheritance.

After the widespread use of various insecticidal products to control *Ae. aegypti*, interest in studying resistance to these compounds began to grow. A study by Bellinato et al. [34] demonstrated significant resistance to both temephos (OP) and deltamethrin (PY) in *Ae. aegypti* populations collected from 12 cities across Brazil between 2010 and 2012. In addition, other non-target species have been impacted by the overuse of these insecticides. For example, a study in Pernambuco state, carried out by Amorim et al. [35], revealed that resistance to temephos had been selected in natural populations of *Cx. quinquefasciatus*, as a consequence of indirect exposure to this larvicide.

According to MoReNAa recommendation, in 2009, two insect growth regulators (IGRs) were introduced in Brazil, the chitin synthesis inhibitors (CSIs) represented by diflubenzuron and novaluron [22,36], and in 2014 a juvenile hormone analogue (JHA), pyriproxyfen, although few wild *Ae. aegypti* populations exhibited decreased susceptibility to this last compound [23]. Simultaneously, malathion was reintroduced for controlling adult *Aedes* mosquitoes, which did not have much effect once dengue cases continued to increase.

In 2020, the Brazilian MoH recommended the use of another larvicide, Spinosad, which is derived from the bacterium *Saccharopolyspora spinosa*. This larvicide has an alternative mode of action compared to many synthetic chemical insecticides. Also, the use of insecticide combinations with different mechanisms of action was recommended for the first time in the country. These included the adulticide Fluodora^®^ (a combination of clothianidin “neonicotinoid”, and deltamethrin PY) for residual spraying, as well as Cielo^®^ (a combination of imidacloprid “neonicotinoid”, and prallethrin PY) for ultra-low volume application [37].

**Figure 2:**
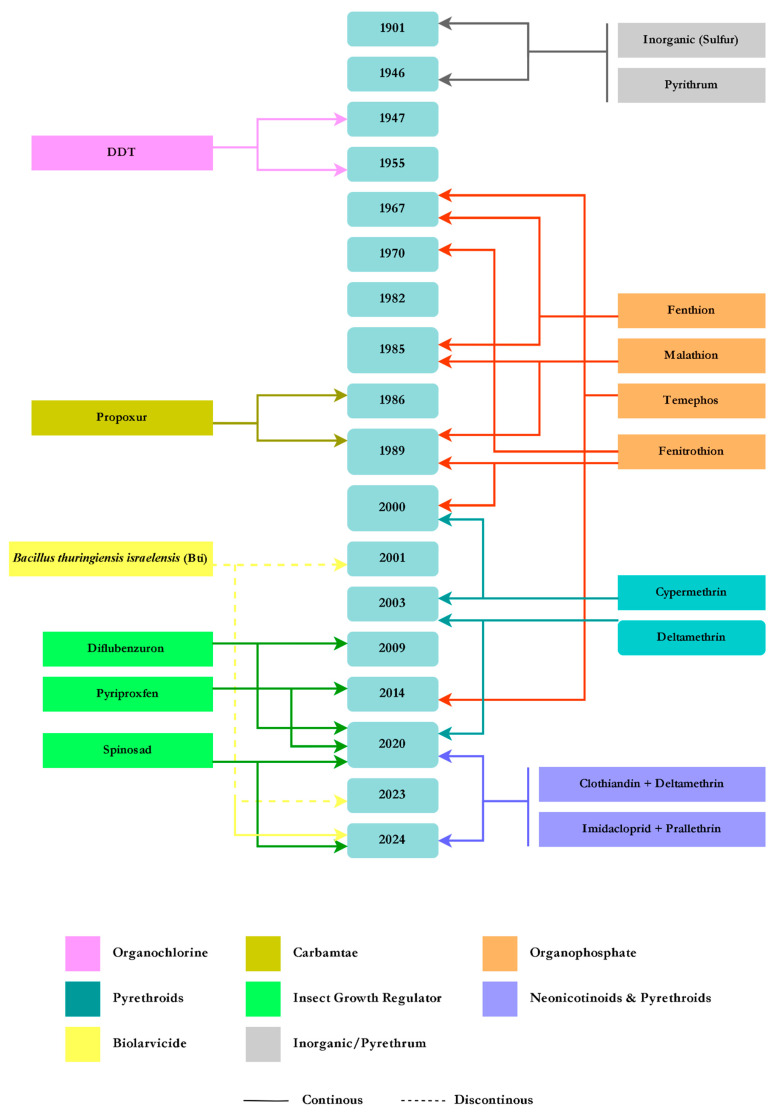
Timeline of the insecticides introduced for *Aedes aegypti* control.

Figure 2 summarizes all the insecticides that have been used up until 2024 to control *Ae. aegypti*.

After the widespread use of various insecticidal products to control *Ae. aegypti*, interest in studying resistance to these compounds began to grow. Montella et al. [27] examined 24 *Ae. aegypti* populations collected between 2002 and 2004, reporting resistance ratios (RR_95_) to temephos ranging from 1.4- to 26.2-fold, with 15 populations exhibiting RR_95_ values >10-fold. These findings already indicated a widespread distribution of resistance across the country after nine years of exclusive use of this larvicide. This cohort study represented a key milestone for the MoReNAa Network, supporting the implementation of resistance-management measures, including the immediate replacement of temephos in field applications and the adoption of additional classes of larvicidal insecticides, such as insect growth regulators (IGRs). A study by Bellinato et al. [34] demonstrated significant resistance to both temephos (OP) and deltamethrin (PY) in *Ae. aegypti* populations collected from 12 cities across Brazil between 2010 and 2012. In addition, other non-target species have been impacted by the overuse of these insecticides. For example, a study in Pernambuco state, carried out by Amorim et al. [35], revealed that resistance mechanisms to temephos had been selected in natural populations of *Cx. quinquefasciatus*.

A study by Valle et al. [38] confirmed the widespread resistance to temephos and deltamethrin in Brazil. Furthermore, Dias et al. [39] investigated the susceptibility of *Ae. aegypti* populations from 46 cities from 2020 to 2023 across Brazil to spinosad (larvicide), Fluodora^®^ (a combination of clothianidin “neonicotinoid”, and deltamethrin PY), and Cielo^®^ (a combination of imidacloprid “neonicotinoid”, and prallethrin PY). The results revealed full susceptibility to larvicide spinosad. However, high to very high resistance to both adulticide formulations was detected. Considering the complexity of the Bti mode of action in Diptera, the risk of resistance selection is low, and in fact, resistance to this biolarvicide in *Ae. aegypti* from Brazil has never been recorded [31], although it is important to highlight that its use was sporadic.

IR, in addition to causing failures and compromising control programs that rely on the use of chemical compounds, has also negatively impacted new strategies for releasing *Wolbachia*-infected (WI) mosquitoes. Because insecticide resistance usually carries a biological cost, (WI) released mosquitoes cannot compete with wild mosquitoes in areas where resistance is widespread. Therefore, in Brazil, resistance had to be incorporated into the production of *Wolbachia*-infected strains [40].

### 3.2. Other Vector Species of Medical Importance

#### 3.2.1. *Anopheles* spp.

Unlike arboviruses like DENV and CHIKV, which have a single main urban mosquito species for which vector control is focused, malaria has several mosquito species involved in its transmission cycle. *Anopheles* species are responsible for transmitting *Plasmodium*, the etiological agent of malaria, which remains a significant health concern in Brazil. Autochthonous *Anopheles* spp. occupy three distinct geographical environments: the Amazon rainforest system, predominantly home to *Anopheles darlingi* Root, 1926, which accounts for the majority of malaria cases in Brazil; the Atlantic rainforest system, where accumulated water in bromeliad plants provides excellent breeding sites for *Anopheles cruzii* Dyar & Knab, 1908 and *Anopheles bellator* Dyar & Knab, 1906; and the Brazilian coastal areas, where *Anopheles aquasalis* Curry, 1932 is the predominant species. Other malaria vectors have been recorded, such as the *Anopheles albitarsis* complex (Lynch Arribálzaga 1878), which includes *Anopheles oryzalimnetes* Wilkerson & Motoki, 2009, *Anopheles deaneorum* Rosa-Freitas, 1989, *Anopheles marajoara*, and *Anopheles janconnae* [41].

Regarding the autochthonous *Anopheles* spp, the first organized prophylactic campaigns against malaria were led by Carlos Chagas in 1905 in Itatinga (São Paulo State) and Baixada Fluminense (Rio de Janeiro State) in 1907. As part of these efforts, an in-house fumigation was carried out using sulfur as an adulticide and petroleum as a larvicide. Additionally, pyrethrum was used as an adulticide alongside the earlier insecticides in Rio de Janeiro [42,43,44].

In addition to the local species, *Anopheles gambiae* Giles, 1902 (later identified as *Anopheles arabiensis* Patton, 1905), a significant invasive species of malaria vector in sub-Saharan Africa, was introduced into Brazil between 1930 and 1940 [42]. Raymond Shannon first documented the presence of *Anopheles gambiae* aquatic stages in 1930 after finding a large number of larvae in a small area subject to flooding in the city of Natal. More than 14,000 people had died as a result of the mosquito’s 1938 spread throughout various regions of the states of Ceará and Rio Grande do Norte [45]. Paris Green was first used as a larvicide in Brazil between 1938 and 1940, in combination with pyrethrum diluted in kerosene as an adulticide. *An. gambiae* was eventually eradicated from northeastern Brazil as a result of these actions [44].

In 1945, DDT was introduced in Pará State, replacing pyrethrum for malaria vector control [42]. From the 1940s to the 1980s, extensive operations were carried out to control *Anopheles* in bromeliad-associated breeding sites, deploying numerous insecticides, including DDT for indoor residual spraying (IRS) and the organophosphate (OP) malathion (outdoor spraying via ultra-low volume), along with larvicides such as copper sulfate and Paris green [42]. However, in 1999, the use of DDT in vector control programs in Brazil was definitively discontinued due to its environmental impact, toxicity, and the development of resistance [46].

In the mid-1980s, pyrethroid (PY) insecticides began to be widely used and continued to be applied in various forms, such as indoor residual spraying against adult *Anopheles*. The use of deltamethrin-impregnated curtains and mosquito nets for malaria control in mining areas of Amapá State was investigated during the 1980s and early 1990s. Studies such as that by Xavier and Lima [47] demonstrated that jute curtains treated with deltamethrin significantly reduced malaria transmission, thereby confirming the effectiveness of insecticide-treated materials in limiting human–vector contact in high-risk, hard-to-reach mining communities. Moreover, in 1992, the residual efficacy of curtains treated with DDT and deltamethrin was evaluated in Amapá, showing significant efficacy for up to 120 days [48].

In Rondônia state, deltamethrin insecticide-treated nets (ITNs) demonstrated a significant reduction in vector density in 1992 [49]. Other PYs, such as cypermethrin WP, lambda-cyhalothrin WP, and etofenprox WP, were evaluated for IRS in Pará state in 2003, with etofenprox showing the highest residual effect, lasting up to four months [50].

In the Amazon region, the use of larvicides is limited due to the difficulty of accessing certain areas, such as mining communities, and concerns about environmental security regarding biodiversity protection. Therefore, the predominant control approach has been the use of adulticides, especially against *An. darlingi*. Since 2009, the Brazilian MH has recommended IRS every three months and the free distribution of insecticide-treated nets ITNs to residences, to use them every night, along with awareness campaigns [51].

From 2008 to 2009, a study by Galardo et al. [52] evaluated *Lysinibacillus sphaericus* as a larvicide in abandoned gold mines in the Amazon rainforest (Amapá state). The larvicides showed significant reductions in both larval and adult stages during the study.

Deltamethrin ITNs were evaluated in 2012, in Rondônia, by Vieira et al. (2014) [53], but no significant results were found. From 2012 to 2014, ITNs impregnated with alpha-cypermethrin and permethrin were evaluated, with a significant reduction in malaria cases observed in the study areas. Alpha-cypermethrin showed more efficacy than permethrin [54].

Since 2011, the Brazilian MoH has officially adopted the use of ITNs as part of the ‘Project on Expansion of Access to Malaria Prevention and Control Measures,’ subsidized by the Global Fund to Fight AIDS, Tuberculosis, and Malaria. As part of this program, 1.1 million ITNs were distributed in priority areas [55]. Currently, lambda-cyhalothrin EC (emulsifiable concentrate) is used as an adulticide via thermal fogging, but only during epidemic situations. The results from Santos et al. [50] led to the replacement of alpha-cypermethrin with etofenprox for IRS applications in 2013 [49].

The literature on insecticide resistance in malaria vectors worldwide, especially in Africa and Asia, is extensive; however, Brazil lacks a structured program for monitoring insecticide resistance in malaria vectors, and studies remain limited. In Amapá, investigations in 2015 and 2019 found *Anopheles darlingi* fully susceptible to PY, while *An. marajoara* showed possible resistance to deltamethrin [56,57]. In Acre, *An. darlingi* populations were resistant to multiple PYs, including etofenprox, deltamethrin, cypermethrin, alpha-cypermethrin, and lambda-cyhalothrin, whereas populations from Pará remained susceptible [58].

**Figure 3:**
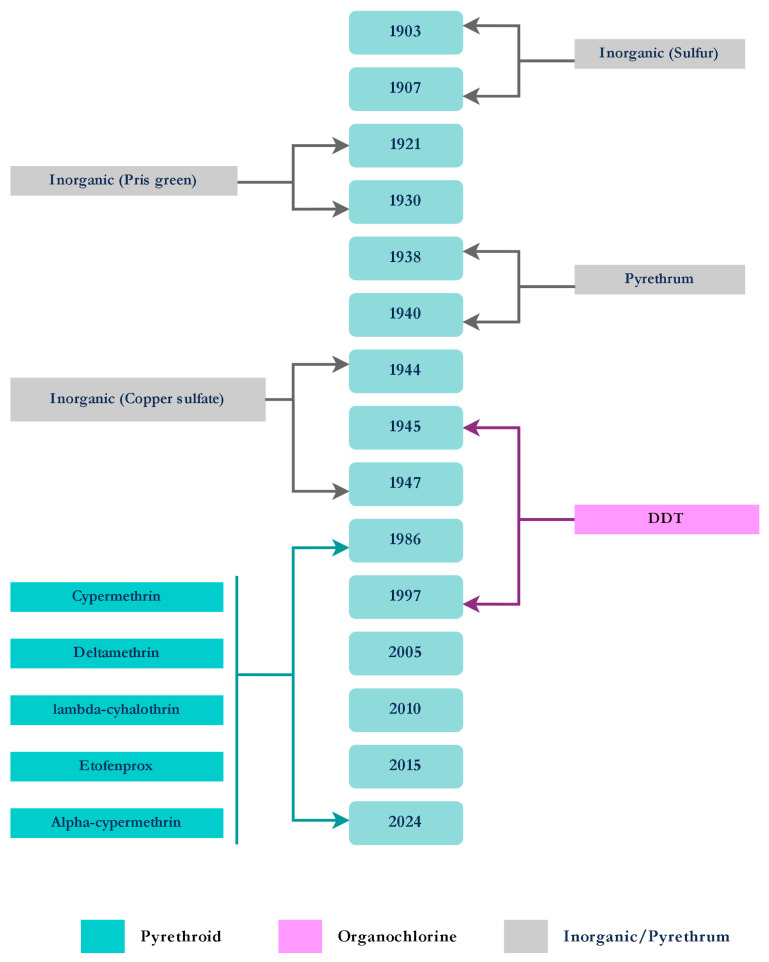
Timeline of the insecticides used for the control of *Anopheles* spp.

From 2021 to 2024, surveys in 19 sites across six Amazonian states applied discriminating concentration (DC) bioassays with deltamethrin, etofenprox, and permethrin. Only four *An. darlingi* populations were fully susceptible to deltamethrin, five to etofenprox, and 11 of 18 tested were susceptible to permethrin. Resistance was widespread in Amazonas and Acre, where most populations showed reduced susceptibility to all three insecticides. Intensity assays at five times the DC (5 × DC) classified resistance as generally low, with one *An. darlingi* population exhibiting moderate resistance [59]. These findings underscore the need to expand monitoring to additional insecticide classes and to investigate underlying resistance mechanisms in *Anopheles* species (Figure 3).

#### 3.2.2. *Culex quinquefasciatus* Say, 1823

In Brazil, unlike the other mosquito species, there are no active national campaigns dedicated to the control of *Cx. quinquefasciatus*, despite being present in all Brazilian cities and being the most common species in the home environment. In addition to its nuisance biting behavior, *Cx. quinquefasciatus* is the primary vector of lymphatic filariasis (LF) [60]. Notably, benzene-hexachloride (BHC) (also known as gamma-hexachlorocyclohexane and Lindane) was used during the first campaign against LF in 1951 [61].

*Lysinibacillus sphaericus* (Lsp) has been utilized in cities such as Recife and São Paulo for control of *Culex* mosquitoes. In pilot studies, (Lsp) was adopted as a larvicide to control the immature stages of *Cx. quinquefasciatus* in Recife [62,63]. Moreover, in trials conducted in Recife, a combination of two biolarvicides, *Lysinibacillus sphaericus* (Lsp) and *Bacillus thuringiensis* var. *israelensis* (Bti), was applied to breeding sites of both *Culex* and *Aedes* mosquitoes. The Bti/Lsp conjugated biolarvicide demonstrated a significant reduction in *Cx. quinquefasciatus* population density [64]. In São Paulo city, this mosquito species is highly prevalent along the Pinheiros River, where it poses a constant nuisance, particularly to residents living near the river. As a result, local authorities implemented mosquito control programs relying on insecticides. Since 1980, malathion and propoxur have been widely applied [65]. Likewise, *Lysinibacillus sphaericus* (Lsp) proved its efficacy against *Cx. quinquefasciatus* larvae in Pinheiros River [66]. The MoH recently recommended spinosyns, bacterial biolarvicides, juvenile hormone analogues (JHAs), and organophosphates for larval control, and pyrethroids and organophosphates for adult control. 

Lopes et al. [67] reviewed the reports of insecticide resistance for *Cx. quinquefasciatus* in Brazil. They obtained very few publications reporting resistance to organophosphates, carbamates, DDT, and pyrethroids in different localities of the country. They concluded that the resistance observed was probably partly the result of the control campaigns targeting *Ae. aegypti* (Figure 2), combined with the widespread use of insecticides by households and private companies. Regarding the biolarvicides (Bti and Lsp), no significant resistance has been reported in *Cx. quinquefasciatus* from Brazil [68].

**Figure 4:**
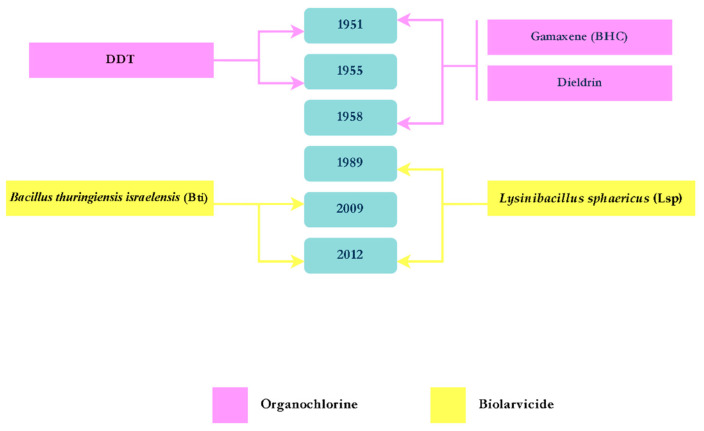
Timeline of the insecticides used for the control of *Culex quinquefasciatus*.

Since the early 2000s, LF endemicity has persisted in only four municipalities in the metropolitan region of Recife: Jaboatão dos Guararapes, Olinda, Paulista, and Recife itself [69,70]. Finally, in 2024, Brazil was certified for the elimination of lymphatic filariasis as a public health problem [71,72]. It is important to highlight that control strategies were a complement to the filariasis elimination program in the city, as the main focus was the mass treatment of the population with DEC (Diethylcarbamazine). The timeline below shows all the insecticides used for the control *Cx. quinquefasciatus* (Figure 4).

#### 3.2.3. Triatomines

Triatomines are the vectors responsible for transmitting Chagas disease. Two tribes (*Triatomini* and *Rhodinini*) include the most important vector species, with *Triatoma infestans* being the most relevant species in Brazil (Klug 1834). Other species of epidemiological importance include *Panstrongylus megistus* (Burmeister 1835), *T. brasiliensis* (Neiva 1911), *T. pseudomaculata* (Corrêa and Espínola 1964), and *T. sordida* (Stål 1859) [73].

The most common methods employed to control the kissing bugs include indoor residual spraying (IRS) and outdoor residual spraying (ORS) using PY. Before 1945, Dias [74] tested a mixture of rotenone and kerosene for indoor spraying, which showed significant results. Interestingly, DDT showed limited effectiveness against most triatomine species, while better results were obtained using other organochlorines such as Dieldrin and Benzene hexachloride (BHC), which were used in both indoor and outdoor residual spraying from the late 1940s until the early 1980s. In the 1980s, pyrethroids were introduced, including cypermethrin, permethrin, cyfluthrin, lambda-cyhalothrin, and deltamethrin [75,76,77,78,79,80,81].

**Figure 5:**
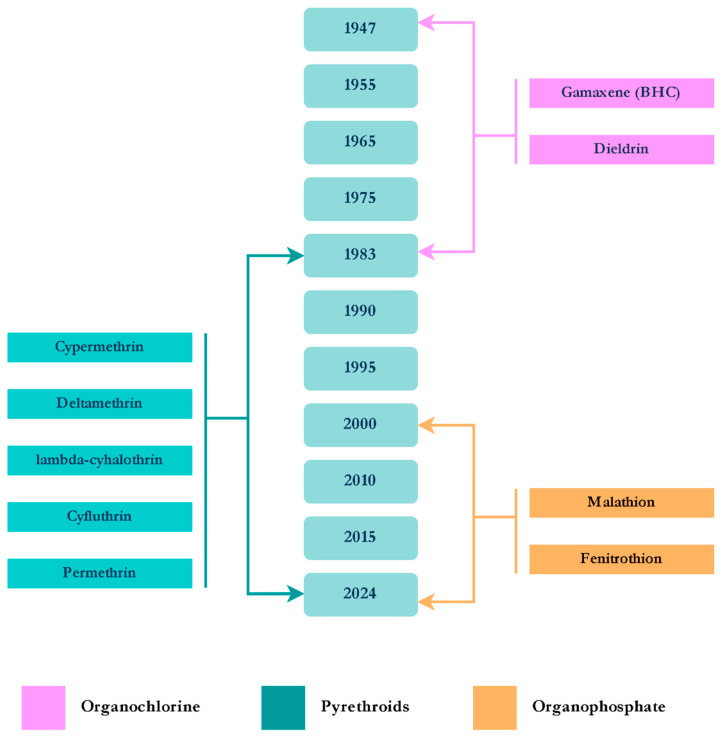
Timeline of the insecticides used for the control of Triatomines.

In Bahia state, malathion (organophosphate) was evaluated in field trials targeting triatomine eggs [82]. Moreover, in the 1980s, Oliveira Filho and his team [83,84] conducted several experiments evaluating alternative insecticides such as bendiocarb and various organophosphates (Chlorpyrifos-ethyl, malathion, and chlorphoxim), as well as pyrethroids like bifenthrin, cyfluthrin, tetramethrin, deltamethrin, prallethrin, esfenvalerate, cyphenothrin, and permethrin [85]. Deltamethrin (pyrethroid), along with malathion (organophosphate), continued to be applied in vector control efforts (Figure 5) [85,86,87]. The timeline below depicts all the insecticides used for triatomine vector control up to 2024 (Figure 5).

Pessoa et al. [88] reviewed the reports of insecticide resistance in triatomine species from 1970 up to 2015. In Brazil, the few reports showed that, in general, triatomine populations have shown low RR (RR_50_ < 8.0). The studies have been focused on the species *T. brasiliensis*, *T. sordida*, *P. megistus*, and *T. infestans* from areas with persistent reinfestations, tested for PY (Deltamethrin and Beta-cyfluthrin).

#### 3.2.4. Phlebotomines

Phlebotomine sand flies are the vectors responsible for transmitting both Visceral Leishmaniasis (VL) and Cutaneous Leishmaniasis (CL). The principal vectors of VL in Brazil are *Lutzomyia longipalpis* (Lutz & Neiva, 1912) and *Lutzomyia cruzi* (Mangabeira 1938). The main vectors involved in CL transmission include *Lutzomyia flaviscutellata* (Mangabeira 1942), *Lu.* (Nyssomyia) *whitmani* (Antunes & Coutinho, 1939), *Lu.* (Nyssomyia) *umbratilis* (Ward & Fraiha 1977), *Lu. intermedia* (Lutz & Neiva 1912), *Lu. wellcomei* (Fraiha, Shaw and Lainson 1971), and *Lu. migonei* (França 1920) [89].

Chemical control of phlebotomine sand flies primarily involves indoor and outdoor residual spraying, targeting adult insects, as the immature stages are extremely difficult to locate in the environment [90]. DDT was first used by Deane and Alencar in 1953 during the initial outbreak of VL in Ceará [91]. Notably, from 1982 to 1993, DDT was used for IRS in Maranhão State, alongside the organophosphate malathion as ULV spraying [92]. Since the mid-1980s, numerous pyrethroids have been deployed, including cypermethrin, etofenprox, lambda-cyhalothrin, and alpha-cypermethrin [93,94,95].

**Figure 6:**
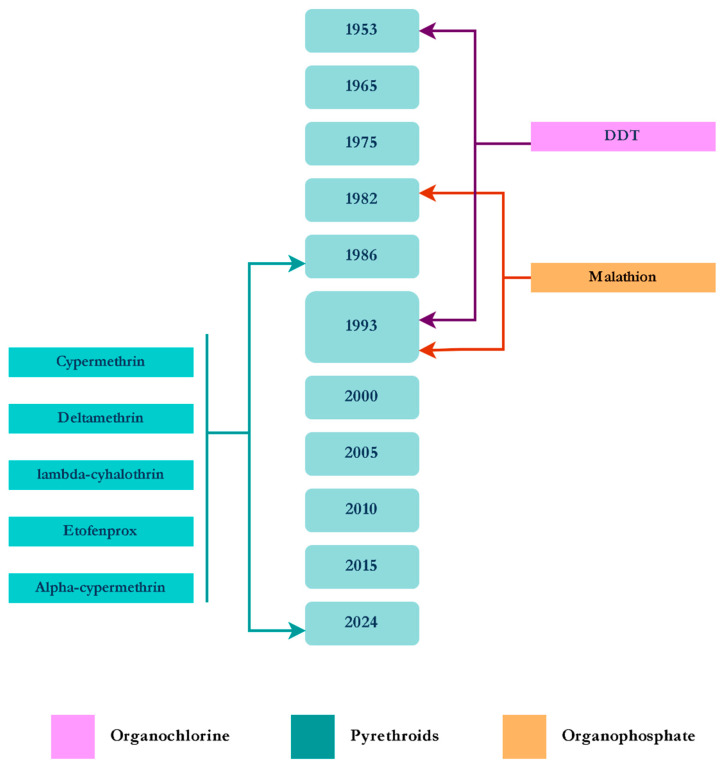
Timeline of the insecticides used for the control of Phlebotomine.

Additionally, pilot studies using deltamethrin-impregnated dog collars (IDCs) have been conducted in São Paulo, Mato Grosso, Ceará, and Minas Gerais States [96,97,98]. A recent study in Minas Gerais evaluated the use of alpha-cypermethrin PY as a lure-and-kill strategy [99]. The timeline below shows all the insecticides used for Phlebotomine vector control up to 2024 (Figure 6).

As with triatomines, only a few papers report insecticide resistance in leishmania vectors in Brazil. They show incipient resistance in *Lutzomyia longipalpis*. Alexander et al. [100] detected the first *Lu. longipalpis* populations in Brazil with reduced susceptibility to the insecticides commonly used to control phlebotomines. de Lima et al. [101] reported an incipient resistance to PY in this species in Ceará and Minas Gerais, from areas that were using insecticide-impregnated dog collars; two out of six exhibited an incipient resistance to deltamethrin, and one showed resistance, while three were fully susceptible. Despite different insecticides being applied (Figure 6), most papers concluded that sand fly populations from Brazil remain susceptible to the most insecticides used so far, including DDT [102,103,104].

**Figure 7:**
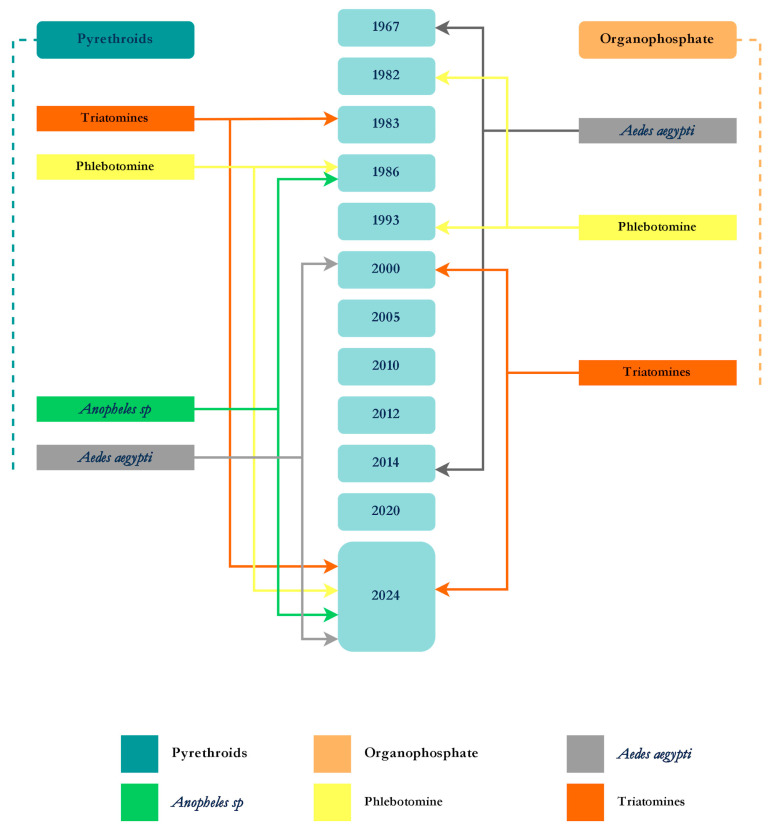
Timeline of the pyrethroids and organophosphates’ overlapping use for control of the four vectors (*Ae. aegypti*, *Anopheles* sp., phlebotomine, and triatomine).

The prolonged use of insecticides for controlling the aforementioned vectors (*Aedes aegypti*, *Anopheles*, Triatomines, and Phlebotomines) has resulted in overlapping applications across different vector species (Figure 7).

## 4. Discussion and Conclusions

**Figure 8:**
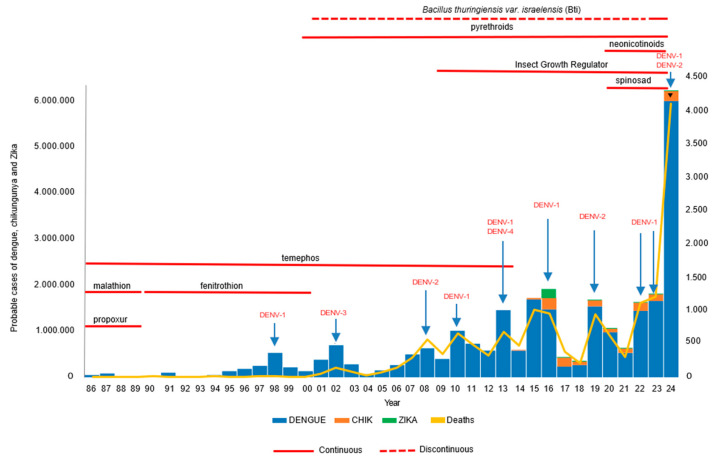
Number of cases of dengue, chikungunya, and Zika over the years, and the use of insecticides for *Ae. aegypti* control [105].

This study showed that all vector control campaigns or vector-borne disease control programs in Brazil have used chemical insecticides from the 1980s to the present. The development of resistance to organophosphate and pyrethroid insecticides, especially in *Aedes aegypti* populations nationwide, has led to their replacement with compounds from alternative classes, such as carbamates and neonicotinoids, or the adoption of formulations combining multiple insecticide classes for adult control [37]. However, dengue cases have been rising since the 1990s and dramatically increased in 2024 [105], showing that chemical insecticide use has not had the expected effect (Figure 8). Nowadays, most Brazilian municipalities are infested by *Ae. aegypti*, and the Brazilian MoH has developed new guidelines for controlling urban arboviruses, focusing on entomological surveillance and vector control [106].

Notably, vector control programs have applied insecticides focused on each target species individually without considering that many cohabitate in the same environment. For example, *Cx. quinquefasciatus* and *Ae. aegypti* in the urban areas. While *Anopheles*’ vector distribution over Brazil is more concentrated in the Amazonian region and the northeastern coast regions [41], it inhabits broad ecological zones alongside other sylvatic vector taxa such as phlebotomine sand flies and triatomines, particularly at forest margins or rural settings [107,108,109,110,111,112,113,114]. Thus, the lack of an integrated species control approach may lead to resistance selection in non-target populations, jeopardizing future control measures. Even within the same program, the amount and duration of insecticide application varied widely across the country, exemplified by the use of organophosphates for *Ae. aegypti* control from 2003 to 2014 [38]. Consequently, this resulted in a partial or complete exposure of these insects to the same insecticide groups in numerous field areas (Figure 7), potentially leading to different insecticide selection pressure. This, in turn, hinders efforts to monitor and manage insecticide resistance. An example is the current situation that challenges the National Dengue Control Program (PNCD), which arose from the widespread resistance of *Ae. aegypti* population to deltamethrin and temephos, as confirmed by recent studies [38,39,40].

For triatomines, most papers show susceptibility to the current insecticides used, and controlling failures have been suggested as the cause of recolonization in the environment after treatments. For such species, as well as for sand flies, there is no permanent program; control measures are carried out through sporadic campaigns, which allow reintroductions from untreated wild areas.

Furthermore, the excessive use of insecticides for vector control imposes considerable economic constraints due to the costly investments in procurement, transportation, and application, especially in large-scale national programs such as PNCD.

The lack of entomological mapping that highlights how insecticides are being used in all vector control actions has hindered an effective response to reduce areas at high risk of transmission. This information, combined with addressing the environmental conditions of the constant presence of vector species, appears to be crucial for the development of sustainable vector control and disease prevention strategies, ultimately improving public health outcomes.

Effective reduction in target species’ population density and, consequently, the incidence of vector-borne diseases (VBDs) requires coordinated and sustained actions. WHO recommends the integrated vector control management (IVM); however, IVM alone is insufficient without a comprehensive understanding of the broader environmental context and the social determinants that underpin the health–disease continuum. These factors are central to the One Health framework, which emphasizes the interconnectedness of human, animal, and environmental health. Brazil’s heavy reliance on chemical insecticides for vector control is a significant issue, as the country is the largest global consumer of pesticides. In 2022, Brazil applied 800.65 thousand metric tons of active ingredients, with 87% imported and many classified as highly hazardous [115,116]. Moreover, the use of insecticides in agricultural areas and for urban pest control, whether by professional services or households, adds a layer of complexity to the regulation and strategic deployment of insecticides in public health.

**Figure 9:**
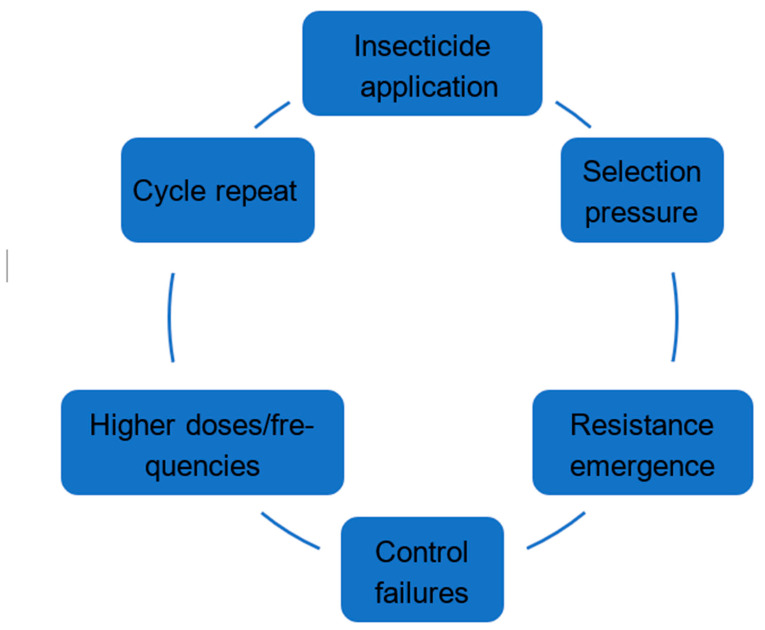
The insecticide resistance treadmill. An escalating cycle of insecticide use and evolving resistance that diminishes long-term control effectiveness.

In summary, the development of high levels of resistance in *Ae. aegypti* populations across all regions of the country reflect the excessive and indiscriminate use of chemical insecticides. This self-perpetuating cycle (insecticide-resistance treadmill) is an expected result, where increasing resistance in insect populations leads to escalating use of higher doses or frequencies of insecticides, which in turn drives even greater resistance (Figure 9), showing that this process is unsustainable; it traps vector control programs in a loop of dependence on chemical control, leading to environmental, health, and economic costs.

Finally, while introducing vaccines for dengue and chikungunya is expected to reduce disease incidence significantly, this does not imply that vector control measures should be discontinued. On the contrary, such efforts should become more comprehensive, better structured, and sustainably integrated, adapted to the environmental context where multiple vector-borne diseases (VBDs) co-occur. The experience accumulated by Brazil can be considered by other countries and used as an example, taking into account the mistakes made and the complexity of the different environments that the country has.

## 5. Future Directions

Considering the results summarized above, it is clear that vector control programs based primarily on the use of chemical insecticides are unsustainable, either due to the development of resistance or the reinfestation of treated areas from wild environments. Additionally, for species for which there is no structured control program, but only temporary campaigns, the implementation of more permanent measures with community participation is recommended. Therefore, it is necessary to consider programs that take environmental management into account, readapting basic infrastructure such as sanitation, ensuring access to permanent drinking water, waste collection, and adequate housing. The development of these programs must be considered within the context of One Health, considering the environment, humans, and animals. In rural areas, where there is no clear separation between human dwellings and the raising of domestic animals or wild species, investment should be made in environmental education, personal protection strategies, and mechanical control methods. These actions should not be designed for a specific program, but rather as an important strategy for all programs.

## Data Availability

No new data were created or analyzed in this study. Data sharing is not applicable to this article.

## Abbreviations

The following abbreviations are used in this manuscript:

BHC	Benzene Hexachloride
BTi	*Bacillus thuringiensis israelensis*
CCl_4_	Carbon Tetrachloride
CL	Cutaneous Leishmaniasis
CSI	Chitin Synthesis Inhibitor
DDT	Dichloro-Diphenyl-Trichloroethane
DEC	Diethylcarbamazine
DENERu	Departamento Nacional de Endemias Rurais (Brazilian National Department for Rural Endemics)
IGR	Insect Growth Regulator
ITN	Insecticide-Treated Net
IVM	Ivermectin
JHA	Juvenile Hormone Analogue
LF	Lymphatic Filariasis
Lsp	*Lysinibacillus sphaericus*
MoReNAa	Monitoramento da Resistência de *Aedes aegypti* a Inseticidas (Brazilian insecticide resistance monitoring network)
MoH	Ministry of Health
OP	Organophosphate
ORS	Outdoor Residual Spraying
PAHO	Pan American Health Organization
PNCD	Programa Nacional de Controle da Dengue (Brazilian National Dengue Control Program)
PY	Pyrethroids
ULV	Ultra-Low Volume
VBD	Vector-Borne Disease
VL	Visceral Leishmaniasis
WHO	World Health Organization
YF	Yellow Fever
